# Promoting Inclusion Oral Health: Social Interventions to Reduce Oral Health Inequities

**DOI:** 10.3390/dj8010005

**Published:** 2020-01-07

**Authors:** Ruth Freeman

**Affiliations:** Dental Health Services Research Unit, Dundee Dental Hospital and School, University of Dundee, Dundee DD1 4HN, UK; r.e.freeman@dundee.ac.uk

**Keywords:** inclusion oral health, social exclusion, homelessness, prisons, undocumented migrants, social and community-based interventions

## Abstract

To advance our understanding of inclusion oral health and to address the impact of social exclusion upon oral health, this group of papers sets out to provide an argument for the need for social and community-based interventions, theoretically underpinned by pluralistic definitions of evidence-based practice and the radical discourse of health promotion for those experiencing exclusion. Using the definition and framework of inclusion oral health, these papers illustrate the requirement for mixed-methods research, the incorporation of experts by experience in the research process, and the need for co-design and co-produced interventions. The papers in this Special Issue present various sources of evidence used to transform top-down into bottom-up community-based interventions for people experiencing homelessness, people in custody, and families residing in areas of high social deprivation. The first two papers provide the evidence for extreme oral health in those experiencing exclusion, and the final four papers report on the implementation and evaluation of social or community-based interventions. This collection of research papers will be of interest to all those wishing to reduce health inequities. This will be achieved by focusing on prevention, adopting a common risk factor agenda, and incorporating co-design and co-production elements into interventions, to tackle the oral health inequities felt by those most excluded in our societies.

## 1. Introduction

The impact of people’s health and disease was recognized in the first Global Burden of Disease report in 2010. Of the fifty nonfatal diseases identified world-wide, four of the diseases were dental caries in adults and children, chronic periodontitis, and edentulous [1]. Not only were dental diseases in the top fifty nonfatal illnesses, but dental caries in adults was tenth in the global burden of disease worldwide [2].

In 2018, Luchenski et al. studied the effect of disease on the most vulnerable populations. This work showed that, while a social gradient existed for the general population, for those experiencing social exclusion, a “so-called cliff-edge of inequality” occurred [3,4], resulting in “extreme health”. To account for such health disparities, Luchenski and her colleagues proposed a definition for inclusion health as the means to “redress health and social inequities among the most vulnerable and marginalized in a community” [1]. 

It was to be another year before a coherent definition for inclusion oral health was available to address the oral health inequities of those “most vulnerable and marginalized” in our societies:

*“Inclusion oral health is based on a theoretically engaged understanding of how social exclusion is produced and experienced, and how forms of exclusion and discrimination intersect to compound oral health outcomes. Inclusion oral health focuses on developing innovative inter-sectoral solutions to tackle the inequities of people enduring extreme oral health”*.

[5]

Underpinned by social exclusion, intersectionality, and othering theory, and the proposition that current dental systems acted as drivers for exclusion rather than inclusion, Freeman et al. [5] postulated a framework to promote oral health inclusion. This framework called for the following: the integration of health and social care policies to drive social justice and reduce prejudice and stigma; and the co-design and co-production of strategies formulated with and for people experiencing exclusion and the planning of “innovative, inter-sectorial services to promote inclusion” [5].

At the core of oral health inclusion were the research methods that provided a platform for experts by experience to contribute, together with the adoption of pluralistic definitions of evidence-based practice [6], to underpin oral health inclusion interventions [6,7,8]. This collection of papers gives the reader a cogent understanding of the role of evidence in the development of social or community-based interventions to promote inclusion oral health. These papers acknowledge the importance of mixed-method research; the role of experts by experience; and the adoption of a common risk factor agenda and the significance of focusing on prevention to tackle inequities experienced by those most excluded in our societies.

## 2. A Synopsis of the Special-Edition Papers

This special issue, entitled “Promoting Inclusion Oral-Health: Social Interventions to Reduce Oral Health Inequities”, has attracted authors who are at the vanguard of inclusion oral health research. The examination of the predictors of oral health in people experiencing homelessness by Beaton et al. [9] and the influence of oral health impacts upon prisoners’ decision to access dental care in prison [10] demonstrates the effect of extreme oral health upon those suffering social exclusion. Beaton et al. [9] and Freeman and Richards [10], therefore, provide the evidence for social interventions based on co-design and co-production strategies to promote oral health, within a common-risk-factor approach, for those experiencing social exclusion.

Returning to the framework of inclusion oral health [5], the papers by Beaton et al. [11] and Rodriguez et al. [12] on homelessness and Lambert [13] and Yuan [14] on undocumented migrants reflect the very essence of inclusion oral health. The requirement for mixed-methods research to include the voice of experts by experience in the research process is no clearer illustrated than in the papers of Beaton et al. [11] and Rodriguez et al. [12]. For Beaton et al. [11] it is the importance of working alliances between service users, health-care professionals, and the Third Sector that are necessary for the successful implementation of community-based programs to promote oral health. For Rodriguez et al. [12] it is the co-design and co-production of oral health and health promotion workshops, using Freire’s [15] critical consciousness, that strengthens social interactions and knowledge transfer. By using oral health as a portal for knowledge development, the young people were able to voice their lived experiences of homelessness, and by doing so, they developed their life skills and their trust in others and strengthened their social interactions [12].

Acknowledging the need for “tuning into people’s universe[s]” [15], Yuan [14] presents the case for cultural diversity for mother–infant community-based oral health improvement programs for undocumented migrants. The success of social support by using the vehicle of oral health intervention [16] is reported here by Yuan. Her work illustrates the importance of working with communities and advancing, “culturally appropriate approach[es] to improve undocumented migrant mothers’ knowledge, attitudes, and self-reported behavior” when caring for their young child’s oral health. The last paper is that by Lambert [13]. Examining the access to care for undocumented migrants, Lambert [13] takes the reader on a journey from extreme oral health to the acceptance of dental treatment. He convincingly shows that working within the system and training social workers as community oral health in an advocate role reduces missed appointments and provides a pathway for the integration of undocumented immigrants into “professional oral health care”.

## 3. Conclusions

The papers in this special edition take the reader on a trajectory from extreme oral health to the co-design and co-production of interventions to tackle the oral health inequities suffered by those experiencing social exclusion. These papers will be of interested to all those who wish to confront oral health inequities, who wish to address “the cliff edge of inequality”, and who wish to promote social justice through the advancement of inclusion oral health.
